# Femoroacetabular Impingement Morphological Changes in Sample of Patients Living in Southern Mexico Using Tomographic Angle Measures

**DOI:** 10.3390/tomography10120141

**Published:** 2024-12-03

**Authors:** Ricardo Cardenas-Dajdaj, Arianne Flores-Rivera, Marcos Rivero-Peraza, Nina Mendez-Dominguez

**Affiliations:** 1 Hospital Regional de Alta Especialidad de la Peninsula de Yucatan, Servicios de Salud del IMSS-BIENESTAR, Merida 97130, Yucatan, Mexico; ricardo_dajdaj@hotmail.com (R.C.-D.); arianne.flores@virtual.uady.mx (A.F.-R.); 00380342@anahuacmayab.edu.mx (M.R.-P.); 2 Facultad de Medicina, Universidad Autónoma de Yucatán, Merida 97000, Yucatan, Mexico; 3 Escuela de Medicina, Universidad Anahuac, Merida 97308, Yucatan, Mexico

**Keywords:** femoroacetabular impingement, prevalence, Mexico, tomography, ambulatory care facilities

## Abstract

Background: Femoroacetabular impingement (FAI) is a condition caused by abnormal contact between the femur head and the acetabulum, which damages the labrum and articular cartilage. While the prevalence and the type of impingement may vary across human groups, the variability among populations with short height or with a high prevalence of overweight has not yet been explored. Latin American studies have rarely been conducted in reference to this condition, including the Mayan and mestizo populations from the Yucatan Peninsula. Objective: We aimed to describe the prevalence of morphological changes in femoroacetabular impingement by measuring radiological angles in abdominopelvic tomography studies in a sample of patients from a population with short height. Methods: In this prospective study, patients with programmed abdominopelvic tomography unrelated to femoroacetabular impingement but with consistent symptoms were included. Among the 98 patients, the overall prevalence of unrelated femoroacetabular impingement was 47%, and the pincer-type was the most frequent. The cam-type occurred more frequently among individuals with taller stature compared to their peers. Alpha and Wiberg angles predicted cam- and pincer-type, respectively, with over 0.95 area under the curve values in ROC analyses. The inter-rater agreement in the study was >91%. Conclusions: In a patient population from Yucatan, Mexico, attending ambulatory consultations unrelated to femoroacetabular impingement, an overall morphological changes prevalence of 47% was observed. Angle measurements using tomographic techniques can be used to predict cam- and pincer-type femoroacetabular impingement. Average stature was observed to be shorter in patients with cam-type femoroacetabular impingement, but body mass index did not vary between groups.

## 1. Introduction

Femoroacetabular impingement (FAI) was first described in a publication by Reinhold Ganz [1], a Swiss orthopedic surgeon, who proposed that certain anatomical variations between the femur and the acetabulum led to abnormal contact, causing early osteoarthritis of the hip. It is possible to define femoroacetabular impingement as a set of signs and symptoms derived from anatomical abnormalities of the femoral head and/or acetabulum that cause abnormal friction between them and which manifests during hip movement—either in flexion or rotation of the hip—which leads to structural damage to the acetabular labrum and articular cartilage [2,3].

The overall prevalence of FAI syndrome is highly variable, as it has been estimated using a wide range of methodologies and in diverse populations, being observed in 3 to 57% of the samples studied, with a discordance according to region, population, and physical activities, while the local prevalence is unknown [4,5]. This is because the methods for assessing and validating prevalence are often missing or only roughly explained. Epidemiological differences in prevalence could undoubtedly indirectly manifest in the anatomical particularities of a specific ethnic group, given that the human species has great phenotypic variation, with intragroup and intergroup patterns according to their ethnic origins, as reflected by anthropometric variables [6].

Given its etiology, FAI can be classified into cam-, pincer-, and mixed-type, depending on the site of anatomical variation. The cam-type is more prevalent in individuals who perform a variety of high-performance sports activities, where the lesion is generated by a repetitive axial load, causing bone overgrowth at the junction of the head with the femoral neck; determination of this type typically requires radiological assessment [6,7].

Pincer-type FAI is commonly characterized by acetabular over-coverage, resulting in compression of the labrum and articular cartilage between the femoral neck and the acetabular border, typically seen in hips that present with deep coxa or acetabular retroversion [8]. The angle of the acetabular version is measured using axial imaging [9,10]; similarly, marginal acetabular bone growths can be observed due to continuous microtrauma and labral degeneration, with later bone metaplasia, which generates over-coverage and is part of the diagnostic findings [11,12,13]. In a high percentage of patients, a mixed pattern, including concomitant cam- and pincer-type morphological changes, is observed.

The population of contemporary Mayan and mestizo descent in Yucatan has been widely characterized as having a shorter stature compared to other American ethnic groups. Although this aspect could be considered as an anatomical peculiarity, it could also be associated clinically and radiographically with the presence, degree, and manifestations of FAI as, in the human body, there are anatomical associations among the different segments, proportional and net circles, diameters, and lengths. In particular, the associations between length or stature and shoulder girdle dimensions by segments and totals have been studied, and it has been found that these associations may be of epidemiological importance [14].

In the contemporary Yucatecan population—both Mayan and mestizo—the authors have described a high prevalence of short and very short stature in the Yucatecan men and women. Furthermore, the body mass index status in the ranges of overweight and obesity is particularly high among the population of Yucatan, with a combined overweight and obesity prevalence of 82.6% for both sexes reported in 2022 [15,16,17]. The degree of overweight/obesity was higher among individuals with a short height [18], as observed in a sample comprising 1424 participants. Although the literature has suggested that there could be an association among anthropometric measurements, radiological measurements, and pincer-type femoroacetabular impingement, the importance and meaning of this possible association is unknown [6]; however, it seems reasonable to consider that, given these phenotypic differences characterizing the Mayan and mestizo population of Yucatan, the prevalence and presentation of FAI may also differ from those of reference human groups. The objective of the present study was to describe the femoroacetabular impingement morphological changes prevalence in a sample of patients from a population with short height, as well as evaluate the reliability of angle measurements for the prediction of FAI.

## 2. Participants and Methods

The inclusion criteria considered patients of both sexes, aged ≥30 years old, who were scheduled for abdominopelvic tomography at the outpatient clinic of the Regional High Specialty Hospital of the Yucatan Peninsula (HRAEPY). Patients were referred from an ambulatory consultation with the medical doctor in charge, with suspected compressive symptoms accompanying pain in one or both hips but had not received a diagnosis of femoroacetabular impingement. Those with imaging findings suggesting a tumor causing femoroacetabular impingement, amputees, or with standing disabilities were excluded. The elimination criteria further included incapability for full visualization of the femoroacetabular joint in tomography. The sample size calculation was obtained using the sample calculation formula for comparison of two proportions:

*n* = (Zα^2^/2 + Zβ)2 × (P1(1 − P1) + P2(1 − P2))/(P1 − P2)^2^,

where Zα^2^/2 is the critical value of the normal distribution in α/2 (95% confidence level, with α of 0.05/2 and critical value of 3.84), Zβ is the critical value of the normal distribution in β (for a power of 80%, the value of β is 0.2 and the critical value is 0.85), P1 is the sample proportion of the first group (patients with FAI findings), and P2 is the sample proportion of the second group (patients without FAI findings). The proposed sample size was 49 participants per group, for a total of 98 [19,20,21].

The main outcome variable was the presence of FAI (dichotomous), along with the type of FAI (cam or pincer). Version, excrescence, calcification, and osteoarthritis were also considered as dependent variables in logistic regression; while the exposure variables were sex (binary) and body mass index and stature as of numeric continuous type.

The tomographic studies were reviewed by two radiologists with training in musculoskeletal imaging between August and October of 2021. Prior to the collection of images, informed consent was obtained, and patients who agreed to participate in the study were asked to complete a sociodemographic data form. Furthermore, anthropometric measurements were obtained using previously described methods—with respect to the Frankfort line as described by Lohman, Roche, and Martorell (1991) [22]—while patients were barefoot, using a portable stadiometer (Seca© 206 (Vogel & Halke, Hamburg, Germany)) and recording the measurements to the nearest millimeter. Weight was measured with a digital scale to the nearest 100 g.

The FAI diagnosis was made through a detailed physical examination, in which the pain was reproduced using two different tests, which simulate the triggering movements, namely, the FADDIR and FABER tests (flexion–adduction/abduction–internal rotation/external rotation). In the first test, flexion was performed in the affected joint at 90°, with adduction and internal rotation; in the second test, flexion was carried out at 90°, with subsequent abduction and external rotation. These tests have a sensitivity of 78% and 60%, respectively, and a specificity of only 10% and 18% prior to tomographic assessment.

Tomographic images were taken using 128 multi-slice CT scanner (Revolution Evo, GE Healthcare, Waukesha, WI, USA) with 64 sections of 1.25 mm thickness, with the patient in a supine position and both lower limbs in parallel. The obtained images were analyzed using AW VolumeShare7 software, performing multiplanar reconstructions and volume rendering, which allowed for the measurement of C-sign, Alpha, Wiberg, and acetabular angles, thus determining morphological changes consistent with FAI and determining its subtypes. Laterality was considered a nominal variable with three possible values: right, left, or both (for cases of left plus right). The radiologists evaluating CT had expertise with MSK, with R.C.D. having five and A.M.B. having eight years of experience.

### 2.1. Statistical Analysis

With respect to the statistical analysis, an anonymized worksheet was used with unique codes for each participant, which was further exported and processed using Stata for Windows version 12 (Stata Corp., Texas College, TX, USA).

Descriptive statistics include frequencies, percentages, and proportions of the general sample and by group. The tables present the means for numerical data and standard deviations as a measure of dispersion with respect to the numerical and categorical variables. For the hypothesis contrast tests, a single-tailed comparison was used; meanwhile, for comparisons between groups (no FAI vs. FAI; no FAI vs. pincer and cam), ANOVA tests were performed.

For the association analysis, logistic regression was performed; models were selected using maximum likelihood closer to 0 and variance explained by pseudo r^2^ values closer to 1 in the range of 0–1. For the analysis, the chosen measure of association was the odds ratio, and the dependent variable was the presence of labral calcification and version, while the independent variables were the presence of left pincer, left cam, right pincer, and right cam. In the regression analysis, odds for ipsilateral labral calcification and version were assessed.

Early osteoarthrosis findings were also included. Finally, a post hoc test was performed, with values above *p >* 0.05 being considered acceptable in the Hosmer–Lemeshow goodness-of-fit test [23], for which a value of *p* > 0.05 was taken as indicating a good fit in the regression models.

Inter-observer agreement was assessed using a kappa test based on the type of FAI. The observed agreement was contrasted against the expected agreement, and significant differences were established when *p <* 0.05; if the observed agreement significantly exceeded the expected agreement, this indicates a better concordance. Finally, the agreement was interpreted as proposed by Landis and Koch, as follows: (a) below 0.0 = Poor; (b) 0.00–0.20 = Slight; (c) 0.21–0.40 = Fair; (d) 0.41–0.60 = Moderate; (e) 0.61–0.80 = Substantial; and (f) 0.81–1.00 = Almost perfect [24].

Receiver operating characteristic (ROC) analyses were performed to assess the area under the curve, and predictor variables were plotted to test the equality of more than two ROC areas, where the explained variables were the type and laterality of FAI and the predictor variables were ipsilateral version and excrescence, along with Alpha and Wiberg angles. The predictive values at cutoff points, including sensitivity and specificity, are presented in the Supplementary Materials.

### 2.2. Ethical Approval

The study was submitted and approved on 4 February 2021, and was carried out in accordance with the Federal Law on the Protection of Personal Data and the Declaration of Helsinki. The Institutional Review Board and Ethics Committee of the Regional High Specialty Hospital of the Yucatan Peninsula approved the protocol in March 2021, with the authorization number 2020-012. Consent was obtained from all the patients prior to participation.

## 3. Results

A total of 98 patients were included, of which 63% were women. The mean general age was 50.8 years. Regarding the sociodemographic characteristics, 80.61% (*n* = 79) of patients were living in the state of Yucatán, while the remaining patients resided elsewhere in the Yucatan peninsula. They all reported being born in the Yucatan, 55.78% (*n* = 55) had at least one Mayan surname, 47.36% (*n* = 46) spoke both Spanish and Mayan, and 50.52% (*n* = 49) only spoke Spanish. The sociodemographic, anthropometric, and tomographic characteristics of the patient population are detailed in Table 1.

**Figure 1:**
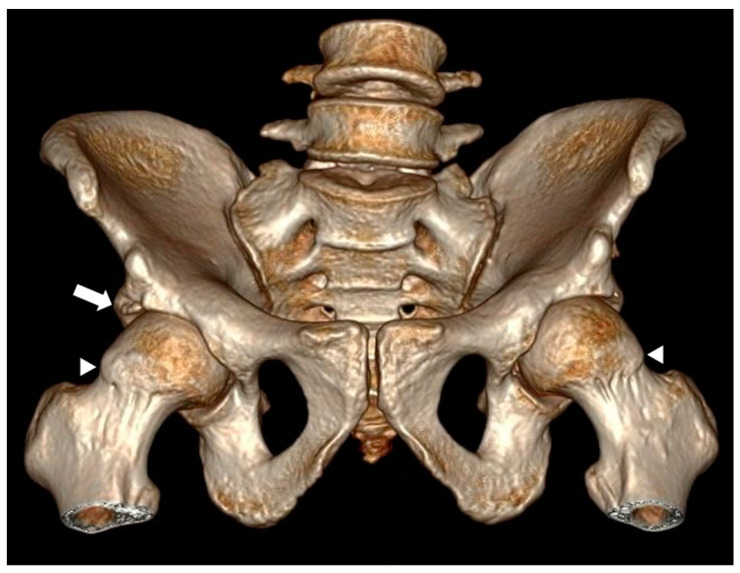
Computed tomography image with 3D volumetric reconstruction showing anterosuperior bony prominence in the cervical–cephalic transition of the bilateral femur, as well as bone excrescence in the right acetabular roof. Findings in relation to right mixed-type and left cam-type femoroacetabular impingement are marked with white arrowheads.

The anthropometric data revealed a general average height of 1.53 m and weight of 68.26 kg (SD ± 14.3). With respect to BMI, the mean was 29 (SD ± 14.3). Of the total patients, 47% presented morphological changes in femoroacetabular impingement, of which 37 correspond to the pincer-type, 17 to the cam-type, and 7 to the mixed-type (an example of mixed-type is shown in Figure 1).

**Figure 2:**
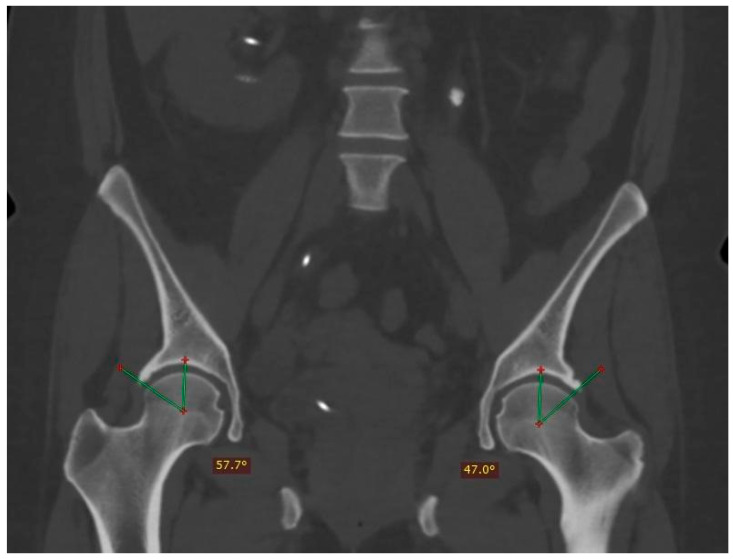
Computed tomography image showing bilateral pincer-type femoroacetabular impingement with coronal reconstruction and window for bone tissues, where acetabular over-coverage was identified with a center-right edge angle of 57.7° and a left angle of 47°. In addition, a stone was observed in the left ureter and a right double J catheter.

The distribution of pincer impingement with respect to laterality was 5.5% on the right side, 16.6% on the left side; while CAM impingement was present in 20% on the left side, 47% on the right, and 33% bilaterally (an example of bilateral FAI is shown in Figure 2).

The cam-type was more prevalent among men, with this type also more commonly occurring among taller individuals, compared with those patients without FAI and those with pincer-type only. Clinical manifestations were significantly more common in patients with cam-type FAI type, while right and left Alpha angles differed between cam-type patients and others.

Wiberg angles had higher amplitude among patients with pincer-type FAI when compared to the overall sample, and labral calcification was also more common among patients with pincer-type FAI. Excrescence (either right or left) and osteoarthritis signs were less prevalent among patients without FAI, when compared to patients with either type of FAI.

Regarding the acetabular version by gender, the right acetabular version in men was 21.085° and, in the left, 20.341°; for women, the right version presented an average of 23.696° and, in the left version, 23.308°.

**Figure 3:**
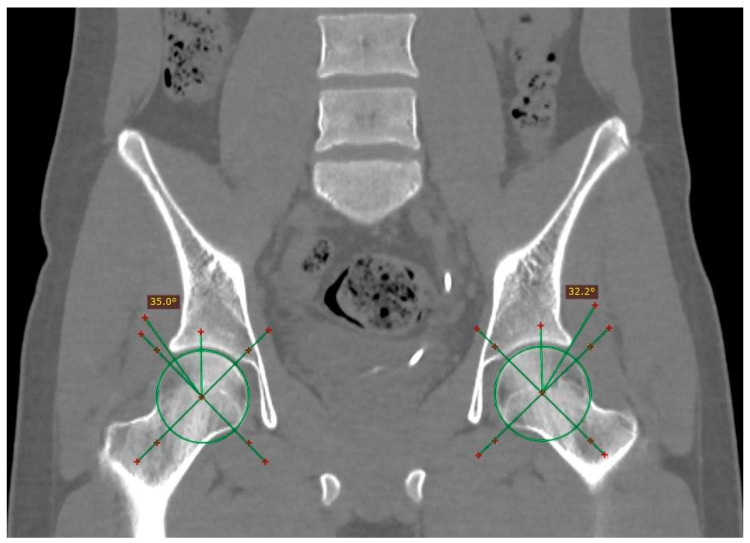
Measurement of the center-edge angle (Wiberg) in an asymptomatic patient. An angle is obtained starting from the center of the femoral head, with a stroke following the vertical axis of the same (90°) and the lateral edge of the acetabulum. The normal value is less than 40°.

Patients who had compressive symptoms more often presented with C-sign, mixed impingement with 16.66% differences in Wiberg’s angle in patients with and without C-sign; furthermore, the average of the angles was greater in patients with C-sign, with a difference of 0.8112 degrees in the right angle and an even more pronounced difference between the averages of the left angle, at 2.7715 degrees (measurement of the Wiberg angle is depicted in Figure 3).

**Figure 4:**
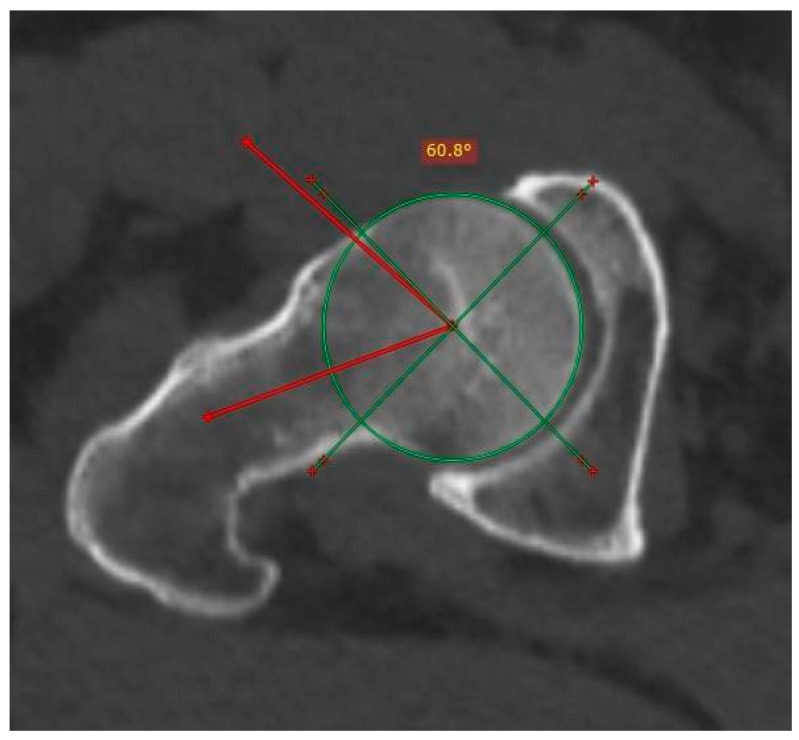
Measurement of the Alpha angle in a patient with femoroacetabular cam impingement. The angle is obtained from the start of the middle femoral head by means of a linear stroke that runs through the center of the femoral neck and another to the point where the sphericity is lost at the junction between the head and the neck. The normal value is equal to or less than 50°.

The Kolmogorov–Smirnoff test confirmed the normal distribution of data (*p* = 0.084). Regarding the interpretation of the mean comparison test, the *p*-values of the variables right Wiberg angle, left Wiberg angle, right excrescence, left excrescence, pincer, and cam suggested significant differences in the frequency of pincer-type FAI among the height groups (Table 1), where the compressing type with alpha angle was more common than non-compressing (Figure 4 depicts the measurement of the Alpha angle).

In the logistic regression results, pincer-type was significantly associated with excrescence but not version; meanwhile, cam-type was not associated with version or excrescence, as detailed in Table 2. The osteoarthritis and labral calcification goodness-of-fit indicated that associations with either pincer- or cam-type FAI were not reliable, as the post hoc test showed *p <* 0.05. Consequently, these variables were omitted.

**Figure 5:**
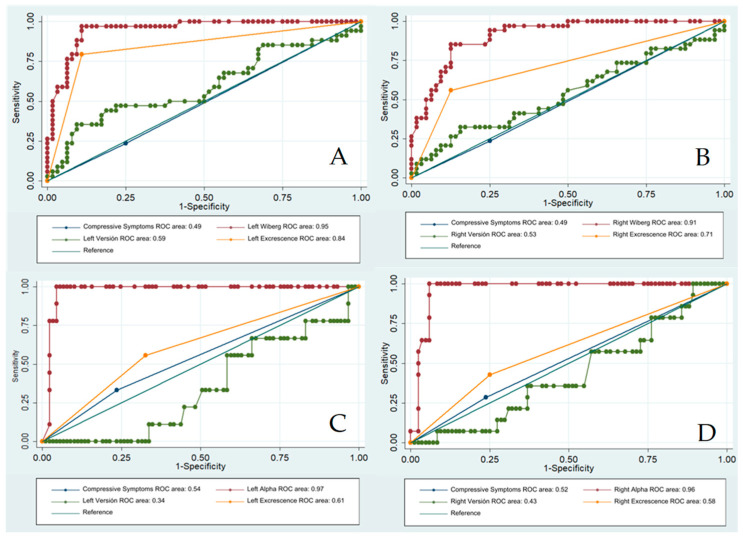
ROC analyses reporting area under the curve values for three predictors of FAI. (**A**) Left Pincer, (**B**) Right Pincer, (**C**) Left Cam, (**D**) Right Cam.

Regarding the receiver operating characteristic (ROC) analyses, Wiberg predicted the presence of the left pincer type with an area under the curve (AUC) of 0.95 ± 0.02 and, on the right side, Wiberg obtained an AUC of 0.96 ± 0.02. The Alpha angle obtained an AUC of 0.97 ± 0.01 for predicting left cam and an AUC of 0.96 ± 0.02 for right cam FAI, as shown in Figure 5. Furthermore, considering version and excrescence AUCs in independent analyses, it was found that excrescence, but not version, may predict ipsilateral pincer-type FAI.

The inter-rater agreement determined using the kappa test showed an average of 0.81, which can be classified as almost perfect. For both types of FAI, the observed agreement significantly exceeded the expected agreement. Nevertheless, pincer-type FAI exhibited greater agreement, as expressed with respect to the observed agreement and kappa variables shown in Table 3.

## 4. Discussion

We studied the prevalence of FAI in a sample of the Mayan–Mestizo population in southern Mexico. The findings revealed that the study population had a shorter stature compared to participants in the other earlier studies [6]. However, no significant association between height and the presence of femoroacetabular impingement was observed; it was only observed that cam-type FAI was more common among individuals with higher stature. Nevertheless, we may also consider this finding with caution, as the cam type is more prevalent in men, and the men in the studied sample were taller than the women on average.

The overall prevalence of morphological changes that predispose individuals to femoroacetabular impingement in the analyzed population varied in relation to that reported in the literature, compared to the work of Zhou et al. [25]. There are also studies reporting a 37% prevalence in asymptomatic athletes. The most recent studies on FAI frequency have been conducted in athlete populations, compared to any other population; as such, studies such as ours conducted in unsuspected and undiagnosed populations are less common. It has been reported that FAI is more common in the Western world when compared to Eastern regions, but detailed references are not very common for non-athlete and undiagnosed populations, and there is a chance that patients from Eastern regions are as affected but remain undiagnosed [26,27]. Although assessing FAI in athletes is critical for their performance, it may be time to start helping patients with different health conditions to be diagnosed and treated, as their quality of life can be improved. With this statement, we do not imply that all patients with morphological changes consistent with FAI should be considered for surgical intervention, as it pertains to the clinician to assess the patients as a whole, considering the symptoms but also their risks, their lifestyle, and most of all, respecting their autonomy by accompanying their patients in a decision-making process. FAI may present intermittently and may be confused with other symptoms in patients with concomitant conditions causing pain, with or without compressive symptoms.

Estimating the prevalence of FAI in an open population using CT is difficult due to the ethical constraints of exposing healthy individuals to ionizing radiation; for this reason, the studies on patients undergoing CT for different reasons are used in the clinical context for estimating the prevalence of FAI, as was recently the case for the study of Bartlett et al., who observed an increased prevalence in patients with acetabular fractures [28]. Furthermore, there exist limitations regarding the use of MRI in an open population, particularly related to the associated costs.

With our study, we hope to raise awareness among health professionals for the consideration of FAI as a concomitant or overlapping cause of pain in their patients as, once a patient is already scheduled for an abdominal CT, it only takes a few more minutes to assess their angle measurements. If diagnosed using CT, further studies may be added, such as MRI.

High-field and high-resolution imaging techniques—particularly 3-Tesla magnetic resonance imaging (3-T MRI)—may be considered the gold standard for FAI diagnosis. We consider that, in patients who are suspected of or diagnosed with FAI, MRI may be the best technique for initial diagnosis and follow-up after interventions or over time. Advancements in high-resolution magnetic resonance imaging (MRI) systems, particularly those with a resolution up to 35 μm, have proven to ensure accuracy in hip assessment and more comfort for patients because no intra-articular injection of contrast media is needed [29]. As the costs for MRI are high for the under-resourced population, CT can be considered as a more affordable option with acceptable sensitivity and specificity, as observed in our study.

We found the proportion of patients with pincer morphology to be higher compared to the figures reported by other centers in the country [6,10]. Meanwhile, the proportion with cam morphology was lower. This finding is consistent with the findings of Albers et al. [8], who observed a higher frequency of anterior femoroacetabular impingement in their case series, a finding that could correspond to the study population, as the cam morphology has been more commonly observed in young subjects who perform high-impact physical activities. The higher proportion of cam-type clamping in men is consistent with the results of the systematic review conducted by Mascarenhas et al. [30].

Our comparisons of acetabular anteversion between men and women—both on the right and left sides—yielded findings similar to those of Ruvalcaba et al. [10] in the Mexican adult population. Gutiérrez-Ramos et al. [6] analyzed the differences in FAI between men and women in a hospital in Mexico City. Their results differed from those reported in the present work, as they identified a higher prevalence of cam-type impingement in men on the right hip; in contrast, in this study, it was the second most prevalent type. Furthermore, the pincer-type predominated in women on the left side in their study, while, in this study, bilateral pincer was the most common FAI.

The prevalence in the present study remains higher than the reported for an adult population from Japan and middle-aged white adults from Canada [31,32].

This study had limitations inherent to risk and cost minimizations. As tomographic studies involve exposure to radiation, the sample size may be considered to be small. The prevalence obtained does not represent the general population in the region, and thus, the generalizability of the results is limited, and they should be considered with caution. Additionally, not all variables that could predispose individuals to FAI were assessed here; for example, occupation, physical activity, and other related characteristics were not assessed. It would be advisable, in future studies, to collect information on the physical activity, occupation, and dietary habits of patients. Our approach to the condition was only conducted from the perspective of diagnostic imaging, enabling a better understanding of clinical context and multidisciplinary related aspects. Long-term follow-up and serial clinical assessments would be useful in order to characterize aspects related to progression.

## 5. Conclusions

In the patient population of Yucatan, Mexico, attending ambulatory consultation unrelated to femoroacetabular impingement, an overall morphological change prevalence of 47% was observed. Angle measurements using tomographic techniques can be used to predict cam- and pincer-type FAI. The average stature was shorter than that observed for patients with cam-type FAI, while the body mass index did not vary among groups. Angle measurement of tomographic images—even when CT is performed for reasons unrelated to femoroacetabular impingement symptoms—can provide reliable diagnoses in symptomatic or symptomatic undiagnosed patients, improving their chances of receiving specific treatment.

## Figures and Tables

**Table 1:** Sociodemographic, anthropometric, and tomographic characteristics of patients in relation to femoroacetabular impingement by type (*n* = 98).

General Sample (*n* = 98)	Mean/Percentage	Impingement (*n* = 47)	Pincer (*n* = 37)	Cam (*n* = 17)
Female %	63.23	55.00	66.66	23.52
Age	50.79	53.03	53.75	50.82
Height	153.01	155.00	152.00	158.76 **
Body Mass Index (Kg/m^2^)	29.08	28.54	28.82	28.96
Weight (Kg)	68.24	68.77	67.59	72.53
Compressive Symptoms	24.29	22.67	22.22	29.41 **
Right Alpha	46.64	46.12	48.15	53.85 **
Left Alpha	45.97	45.56	46.88	51.02 **
Right Wiberg	38.67	42.11	44.61 *	37.70
Left Wiberg	40.05	44.04	46.54 *	37.77
Right Version	22.68	23.03	22.16	21.26
Left Version	22.16	23.16	21.67	20.59
Right Excrescence	27.55 *	49.46	51.61	41.17
Left Excrescence	34.69 *	55.68	58.06	47.05
Labral Calcification	30.61	29.03	24.3 **	29.41
Osteoarthritis Signs	12.24 **	25.80	16.21	23.52

* Significantly different at *p* < 0.05; ** Significantly different at *p* < 0.01.

**Table 2:** Logistic regressions of the independent variables, adjusted by groups, as a function of femoroacetabular impingement (*n* = 96).

Type and Laterality	Odds Ratio	Std. Err.	z	*p* > z	[95% Confidence Interval]	
Left Pincer						
Left Version	1.10	0.06	1.77	0.08	0.99	1.21
Left Excrescence	36.34	22.60	5.78	0.00	10.75	122.93
Left Cam						
Left Version	0.91	0.06	−1.46	0.15	0.81	1.03
Left Excrescence	2.60	1.87	1.33	0.18	0.64	10.64
Right Cam						
Right Version	0.97	0.05	−0.67	0.50	0.87	1.07
Right Excrescence	2.26	1.35	1.37	0.17	0.70	7.30
Right Pincer						
Right Version	1.04	0.05	0.70	0.48	0.94	1.15
Right Excrescence	24.55	14.48	5.43	0.00	7.73	78.03

**Table 3:** Inter-rater agreement for identifying femoroacetabular impingement (FAI) by type in a sample of 98 patients.

FAI Type	Radiologist A	Radiologist B	Observed Agreement	Expected Agreement	Kappa	Standard Error	z	*p*	Interpretation
Cam	37	36	91.84%	76.66%	0.65	0.09	6.87	0.000	Substantial
Pincer	16	18	96.94%	53.75%	0.93	0.10	9.26	0.000	Almost Perfect
Overall	53	54	94.39%	88.13%	0.81	0.98	8.10	0.000	Almost Perfect

## Data Availability

Databases can be found at the ResearchGate profiles of the first and corresponding authors.

## Supplementary Materials

The following supporting information can be downloaded at: https://www.mdpi.com/article/10.3390/tomography10120141/s1, Table S1: tables with ROC analysis indicating specificity and sensitivity at different cutoff points.
