# Supplementary material for: Femoroacetabular Impingement Morphological Changes in Sample of Patients Living in Southern Mexico Using Tomographic Angle Measures
Source: Tomography. 2024 Dec 3;10(12):1947–58. doi: 10.3390/tomography10120141 (PMC11678971; doi:10.3390/tomography10120141)
Supplement: Supplementary file 1 [file tomography-10-00141-s001.zip › tomography-3223139-supplementary.pdf]

```
  ___  ____  ____  ____  ____(R)
 /__    /   ____/   /   ____/
___/   /   /___/   /   /___/
  Statistics/Data Analysis
```

```
  ___  ____  ____  ____  ____ (R)
 /__    /   ____/   /   ____/
___/   /   /___/   /   /___/
  Statistics/Data Analysis
```

Copyright 1985-2011 StataCorp LP
StataCorp
4905 Lakeway Drive
College Station, Texas 77845 USA
800-STATA-PC   http://www.stata.com
979-696-4600   stata@stata.com
979-696-4601 (fax)

Serial number:
Licensed to:

Notes:

1 . use

2 . roctab Left_Pincer Left_Wiberg, detail

Detailed report of sensitivity and specificity

Cutpoint	Sensitivity	Specificity	Correctly Classified	LR+	LR-
(>= 22.9)	100.00%	0.00%	34.69%	1.0000	
(>= 28.3)	100.00%	1.56%	35.71%	1.0159	0.0000
(>= 29.8)	100.00%	3.13%	36.73%	1.0323	0.0000
(>= 29.9)	100.00%	4.69%	37.76%	1.0492	0.0000
(>= 30)	100.00%	6.25%	38.78%	1.0667	0.0000
(>= 30.5)	100.00%	7.81%	39.80%	1.0847	0.0000
(>= 30.8)	100.00%	9.38%	40.82%	1.1034	0.0000
(>= 31)	100.00%	10.94%	41.84%	1.1228	0.0000
(>= 31.1)	100.00%	12.50%	42.86%	1.1429	0.0000
(>= 31.8)	100.00%	14.06%	43.88%	1.1636	0.0000
(>= 32)	100.00%	15.63%	44.90%	1.1852	0.0000
(>= 32.4)	100.00%	20.31%	47.96%	1.2549	0.0000
(>= 32.6)	100.00%	21.88%	48.98%	1.2800	0.0000
(>= 32.9)	100.00%	23.44%	50.00%	1.3061	0.0000
(>= 33)	100.00%	25.00%	51.02%	1.3333	0.0000
(>= 34)	100.00%	28.13%	53.06%	1.3913	0.0000
(>= 34.2)	100.00%	29.69%	54.08%	1.4222	0.0000
(>= 34.4)	100.00%	31.25%	55.10%	1.4545	0.0000
(>= 34.7)	100.00%	32.81%	56.12%	1.4884	0.0000
(>= 34.9)	100.00%	34.38%	57.14%	1.5238	0.0000
(>= 35.5)	100.00%	35.94%	58.16%	1.5610	0.0000
(>= 35.6)	100.00%	37.50%	59.18%	1.6000	0.0000
(>= 36)	100.00%	42.19%	62.24%	1.7297	0.0000
(>= 36.2)	100.00%	45.31%	64.29%	1.8286	0.0000
(>= 36.5)	100.00%	46.88%	65.31%	1.8824	0.0000
(>= 36.6)	100.00%	51.56%	68.37%	2.0645	0.0000
(>= 36.7)	100.00%	53.13%	69.39%	2.1333	0.0000
(>= 36.8)	100.00%	54.69%	70.41%	2.2069	0.0000
(>= 37)	100.00%	57.81%	72.45%	2.3704	0.0000
(>= 37.1)	97.06%	59.38%	72.45%	2.3891	0.0495
(>= 37.2)	97.06%	60.94%	73.47%	2.4847	0.0483
(>= 37.4)	97.06%	62.50%	74.49%	2.5882	0.0471
(>= 37.7)	97.06%	64.06%	75.51%	2.7008	0.0459
(>= 37.8)	97.06%	65.63%	76.53%	2.8235	0.0448
(>= 38)	97.06%	67.19%	77.55%	2.9580	0.0438
(>= 38.1)	97.06%	68.75%	78.57%	3.1059	0.0428
(>= 38.3)	97.06%	70.31%	79.59%	3.2693	0.0418
(>= 38.5)	97.06%	71.88%	80.61%	3.4510	0.0409
(>= 38.9)	97.06%	75.00%	82.65%	3.8824	0.0392
(>= 39)	97.06%	76.56%	83.67%	4.1412	0.0384

(>= 39.4)	97.06%	78.13%	84.69%	4.4370	0.0376
(>= 39.6)	97.06%	79.69%	85.71%	4.7783	0.0369
(>= 39.7)	97.06%	81.25%	86.73%	5.1765	0.0362
(>= 39.8)	97.06%	84.38%	88.78%	6.2118	0.0349
(>= 40.3)	97.06%	85.94%	89.80%	6.9020	0.0342
(>= 40.7)	97.06%	87.50%	90.82%	7.7647	0.0336
(>= 40.8)	97.06%	89.06%	91.84%	8.8739	0.0330
(>= 40.9)	94.12%	89.06%	90.82%	8.6050	0.0660
(>= 41)	88.24%	89.06%	88.78%	8.0672	0.1321
(>= 41.2)	88.24%	90.63%	89.80%	9.4118	0.1298
(>= 41.6)	85.29%	90.63%	88.78%	9.0980	0.1623
(>= 41.8)	79.41%	90.63%	86.73%	8.4706	0.2272
(>= 42.1)	79.41%	92.19%	87.76%	10.1647	0.2233
(>= 42.4)	76.47%	92.19%	86.73%	9.7882	0.2552
(>= 42.7)	76.47%	93.75%	87.76%	12.2353	0.2510
(>= 42.9)	73.53%	93.75%	86.73%	11.7647	0.2824
(>= 43.7)	70.59%	93.75%	85.71%	11.2941	0.3137
(>= 43.9)	67.65%	93.75%	84.69%	10.8235	0.3451
(>= 44)	64.71%	93.75%	83.67%	10.3529	0.3765
(>= 44.7)	61.76%	93.75%	82.65%	9.8824	0.4078
(>= 45.5)	58.82%	93.75%	81.63%	9.4118	0.4392
(>= 45.7)	58.82%	95.31%	82.65%	12.5490	0.4320
(>= 45.9)	58.82%	96.88%	83.67%	18.8235	0.4250
(>= 46)	55.88%	96.88%	82.65%	17.8824	0.4554
(>= 46.3)	50.00%	98.44%	81.63%	32.0000	0.5079
(>= 47)	47.06%	98.44%	80.61%	30.1176	0.5378
(>= 47.2)	44.12%	98.44%	79.59%	28.2353	0.5677
(>= 47.3)	41.18%	98.44%	78.57%	26.3529	0.5976
(>= 47.4)	38.24%	98.44%	77.55%	24.4706	0.6275
(>= 48.4)	35.29%	98.44%	76.53%	22.5882	0.6573
(>= 49)	32.35%	98.44%	75.51%	20.7059	0.6872
(>= 49.8)	29.41%	98.44%	74.49%	18.8235	0.7171
(>= 50)	26.47%	98.44%	73.47%	16.9412	0.7470
(>= 50.8)	26.47%	100.00%	74.49%		0.7353
(>= 51)	23.53%	100.00%	73.47%		0.7647
(>= 51.9)	20.59%	100.00%	72.45%		0.7941
(>= 52)	17.65%	100.00%	71.43%		0.8235
(>= 52.1)	14.71%	100.00%	70.41%		0.8529
(>= 57)	11.76%	100.00%	69.39%		0.8824
(>= 58)	8.82%	100.00%	68.37%		0.9118
(>= 61)	5.88%	100.00%	67.35%		0.9412
(>= 63)	2.94%	100.00%	66.33%		0.9706
(> 63)	0.00%	100.00%	65.31%		1.0000

Obs	ROC Area	Std. Err.	—Asymptotic Normal— [95% Conf.	Interval]
98	0.9506	0.0207	0.90993	0.99126

3 . roctab Right_Pincer Right_Wiberg, detail

Detailed report of sensitivity and specificity

Cutpoint	Sensitivity	Specificity	Correctly Classified	LR+	LR-
(>= 25)	100.00%	0.00%	30.61%	1.0000	
(>= 26.9)	100.00%	1.47%	31.63%	1.0149	0.0000
(>= 28)	100.00%	2.94%	32.65%	1.0303	0.0000
(>= 29.4)	100.00%	7.35%	35.71%	1.0794	0.0000
(>= 30.2)	100.00%	8.82%	36.73%	1.0968	0.0000
(>= 30.4)	100.00%	10.29%	37.76%	1.1148	0.0000
(>= 30.6)	100.00%	14.71%	40.82%	1.1724	0.0000
(>= 31)	100.00%	17.65%	42.86%	1.2143	0.0000
(>= 31.1)	100.00%	19.12%	43.88%	1.2364	0.0000

(>= 31.4)	100.00%	20.59%	44.90%	1.2593	0.0000
(>= 31.8)	100.00%	23.53%	46.94%	1.3077	0.0000
(>= 32)	100.00%	25.00%	47.96%	1.3333	0.0000
(>= 32.1)	100.00%	26.47%	48.98%	1.3600	0.0000
(>= 32.2)	100.00%	29.41%	51.02%	1.4167	0.0000
(>= 33.4)	100.00%	30.88%	52.04%	1.4468	0.0000
(>= 33.5)	100.00%	32.35%	53.06%	1.4783	0.0000
(>= 33.8)	100.00%	33.82%	54.08%	1.5111	0.0000
(>= 34)	100.00%	35.29%	55.10%	1.5455	0.0000
(>= 34.4)	100.00%	36.76%	56.12%	1.5814	0.0000
(>= 34.7)	100.00%	38.24%	57.14%	1.6190	0.0000
(>= 34.9)	100.00%	39.71%	58.16%	1.6585	0.0000
(>= 35)	100.00%	41.18%	59.18%	1.7000	0.0000
(>= 35.1)	100.00%	44.12%	61.22%	1.7895	0.0000
(>= 35.6)	100.00%	45.59%	62.24%	1.8378	0.0000
(>= 35.8)	100.00%	47.06%	63.27%	1.8889	0.0000
(>= 36)	100.00%	48.53%	64.29%	1.9429	0.0000
(>= 36.5)	100.00%	50.00%	65.31%	2.0000	0.0000
(>= 36.6)	100.00%	51.47%	66.33%	2.0606	0.0000
(>= 36.7)	100.00%	52.94%	67.35%	2.1250	0.0000
(>= 36.9)	100.00%	55.88%	69.39%	2.2667	0.0000
(>= 37)	100.00%	57.35%	70.41%	2.3448	0.0000
(>= 37.2)	100.00%	61.76%	73.47%	2.6154	0.0000
(>= 37.5)	100.00%	64.71%	75.51%	2.8333	0.0000
(>= 37.6)	100.00%	66.18%	76.53%	2.9565	0.0000
(>= 37.7)	100.00%	67.65%	77.55%	3.0909	0.0000
(>= 38)	100.00%	69.12%	78.57%	3.2381	0.0000
(>= 38.2)	100.00%	72.06%	80.61%	3.5789	0.0000
(>= 38.4)	100.00%	73.53%	81.63%	3.7778	0.0000
(>= 38.5)	100.00%	75.00%	82.65%	4.0000	0.0000
(>= 38.7)	100.00%	76.47%	83.67%	4.2500	0.0000
(>= 39)	100.00%	79.41%	85.71%	4.8571	0.0000
(>= 39.3)	100.00%	86.76%	90.82%	7.5556	0.0000
(>= 39.6)	100.00%	88.24%	91.84%	8.5000	0.0000
(>= 39.7)	100.00%	89.71%	92.86%	9.7143	0.0000
(>= 40.2)	100.00%	91.18%	93.88%	11.3333	0.0000
(>= 40.9)	90.00%	91.18%	90.82%	10.2000	0.1097
(>= 41)	86.67%	91.18%	89.80%	9.8222	0.1462
(>= 41.2)	86.67%	92.65%	90.82%	11.7867	0.1439
(>= 41.4)	83.33%	92.65%	89.80%	11.3333	0.1799
(>= 41.6)	80.00%	92.65%	88.78%	10.8800	0.2159
(>= 41.8)	76.67%	92.65%	87.76%	10.4267	0.2519
(>= 41.9)	73.33%	92.65%	86.73%	9.9733	0.2878
(>= 42)	70.00%	94.12%	86.73%	11.9000	0.3188
(>= 42.1)	66.67%	94.12%	85.71%	11.3333	0.3542
(>= 42.3)	66.67%	95.59%	86.73%	15.1111	0.3487
(>= 42.5)	63.33%	95.59%	85.71%	14.3556	0.3836
(>= 42.7)	60.00%	95.59%	84.69%	13.6000	0.4185
(>= 43)	60.00%	97.06%	85.71%	20.4000	0.4121
(>= 44)	53.33%	97.06%	83.67%	18.1333	0.4808
(>= 45.9)	50.00%	97.06%	82.65%	17.0000	0.5152
(>= 46.1)	46.67%	97.06%	81.63%	15.8667	0.5495
(>= 47)	46.67%	98.53%	82.65%	31.7333	0.5413
(>= 47.2)	43.33%	98.53%	81.63%	29.4667	0.5751
(>= 48.2)	40.00%	98.53%	80.61%	27.2000	0.6090
(>= 49)	36.67%	98.53%	79.59%	24.9333	0.6428
(>= 50.1)	30.00%	100.00%	78.57%		0.7000
(>= 50.5)	26.67%	100.00%	77.55%		0.7333
(>= 51)	23.33%	100.00%	76.53%		0.7667
(>= 51.2)	13.33%	100.00%	73.47%		0.8667
(>= 53)	10.00%	100.00%	72.45%		0.9000
(>= 54)	6.67%	100.00%	71.43%		0.9333
(>= 55)	3.33%	100.00%	70.41%		0.9667
(> 55)	0.00%	100.00%	69.39%		1.0000

Obs	ROC Area	Std. Err.	—Asymptotic Normal— [95% Conf.	Interval]
98	0.9654	0.0162	0.93378	0.99710

4 . roctab Left_Cam Left_Alpha, detail

Detailed report of sensitivity and specificity

Cutpoint	Sensitivity	Specificity	Correctly Classified	LR+	LR-
(>= 36.2)	100.00%	0.00%	9.18%	1.0000	
(>= 37)	100.00%	1.12%	10.20%	1.0114	0.0000
(>= 37.9)	100.00%	2.25%	11.22%	1.0230	0.0000
(>= 38)	100.00%	3.37%	12.24%	1.0349	0.0000
(>= 38.7)	100.00%	6.74%	15.31%	1.0723	0.0000
(>= 39)	100.00%	7.87%	16.33%	1.0854	0.0000
(>= 39.8)	100.00%	10.11%	18.37%	1.1125	0.0000
(>= 40)	100.00%	12.36%	20.41%	1.1410	0.0000
(>= 40.4)	100.00%	14.61%	22.45%	1.1711	0.0000
(>= 40.5)	100.00%	15.73%	23.47%	1.1867	0.0000
(>= 40.6)	100.00%	16.85%	24.49%	1.2027	0.0000
(>= 41)	100.00%	19.10%	26.53%	1.2361	0.0000
(>= 41.2)	100.00%	21.35%	28.57%	1.2714	0.0000
(>= 41.7)	100.00%	22.47%	29.59%	1.2899	0.0000
(>= 41.8)	100.00%	23.60%	30.61%	1.3088	0.0000
(>= 42)	100.00%	24.72%	31.63%	1.3284	0.0000
(>= 42.3)	100.00%	26.97%	33.67%	1.3692	0.0000
(>= 42.6)	100.00%	28.09%	34.69%	1.3906	0.0000
(>= 43)	100.00%	29.21%	35.71%	1.4127	0.0000
(>= 43.5)	100.00%	31.46%	37.76%	1.4590	0.0000
(>= 44)	100.00%	33.71%	39.80%	1.5085	0.0000
(>= 44.4)	100.00%	38.20%	43.88%	1.6182	0.0000
(>= 44.8)	100.00%	39.33%	44.90%	1.6481	0.0000
(>= 45)	100.00%	40.45%	45.92%	1.6792	0.0000
(>= 45.3)	100.00%	48.31%	53.06%	1.9348	0.0000
(>= 45.4)	100.00%	49.44%	54.08%	1.9778	0.0000
(>= 45.6)	100.00%	50.56%	55.10%	2.0227	0.0000
(>= 45.7)	100.00%	53.93%	58.16%	2.1707	0.0000
(>= 45.8)	100.00%	56.18%	60.20%	2.2821	0.0000
(>= 45.9)	100.00%	57.30%	61.22%	2.3421	0.0000
(>= 46)	100.00%	58.43%	62.24%	2.4054	0.0000
(>= 46.1)	100.00%	64.04%	67.35%	2.7812	0.0000
(>= 46.2)	100.00%	65.17%	68.37%	2.8710	0.0000
(>= 46.6)	100.00%	66.29%	69.39%	2.9667	0.0000
(>= 46.8)	100.00%	67.42%	70.41%	3.0690	0.0000
(>= 47)	100.00%	70.79%	73.47%	3.4231	0.0000
(>= 47.5)	100.00%	75.28%	77.55%	4.0455	0.0000
(>= 47.7)	100.00%	77.53%	79.59%	4.4500	0.0000
(>= 47.8)	100.00%	78.65%	80.61%	4.6842	0.0000
(>= 48)	100.00%	79.78%	81.63%	4.9444	0.0000
(>= 48.3)	100.00%	84.27%	85.71%	6.3571	0.0000
(>= 48.4)	100.00%	86.52%	87.76%	7.4167	0.0000
(>= 48.5)	100.00%	87.64%	88.78%	8.0909	0.0000
(>= 48.8)	100.00%	89.89%	90.82%	9.8889	0.0000
(>= 49)	100.00%	91.01%	91.84%	11.1250	0.0000
(>= 49.3)	100.00%	92.13%	92.86%	12.7143	0.0000
(>= 49.6)	100.00%	93.26%	93.88%	14.8333	0.0000
(>= 50.8)	100.00%	94.38%	94.90%	17.8000	0.0000
(>= 50.9)	100.00%	95.51%	95.92%	22.2500	0.0000
(>= 52)	88.89%	95.51%	94.90%	19.7778	0.1163
(>= 52.4)	77.78%	95.51%	93.88%	17.3055	0.2327
(>= 53.2)	77.78%	96.63%	94.90%	23.0741	0.2300
(>= 56)	77.78%	97.75%	95.92%	34.6111	0.2273
(>= 57)	55.56%	97.75%	93.88%	24.7222	0.4547
(>= 57.2)	44.44%	97.75%	92.86%	19.7778	0.5683

(>= 59.4)	33.33%	97.75%	91.84%	14.8333	0.6820
(>= 60)	11.11%	97.75%	89.80%	4.9444	0.9093
(> 60)	0.00%	100.00%	90.82%		1.0000

Obs	ROC Area	Std. Err.	Asymptotic Normal [95% Conf. Interval]	
98	0.9738	0.0157	0.94300	1.00000

5 . roctab Right_Cam Right_Alpha, detail

Detailed report of sensitivity and specificity

Cutpoint	Sensitivity	Specificity	Correctly Classified	LR+	LR-
(>= 30)	100.00%	0.00%	14.29%	1.0000	
(>= 35.6)	100.00%	1.19%	15.31%	1.0120	0.0000
(>= 36)	100.00%	2.38%	16.33%	1.0244	0.0000
(>= 36.6)	100.00%	3.57%	17.35%	1.0370	0.0000
(>= 37)	100.00%	4.76%	18.37%	1.0500	0.0000
(>= 37.7)	100.00%	5.95%	19.39%	1.0633	0.0000
(>= 37.8)	100.00%	7.14%	20.41%	1.0769	0.0000
(>= 38.1)	100.00%	8.33%	21.43%	1.0909	0.0000
(>= 38.5)	100.00%	9.52%	22.45%	1.1053	0.0000
(>= 38.8)	100.00%	11.90%	24.49%	1.1351	0.0000
(>= 38.9)	100.00%	13.10%	25.51%	1.1507	0.0000
(>= 39)	100.00%	14.29%	26.53%	1.1667	0.0000
(>= 39.5)	100.00%	16.67%	28.57%	1.2000	0.0000
(>= 40.3)	100.00%	17.86%	29.59%	1.2174	0.0000
(>= 41)	100.00%	19.05%	30.61%	1.2353	0.0000
(>= 41.3)	100.00%	20.24%	31.63%	1.2537	0.0000
(>= 41.4)	100.00%	21.43%	32.65%	1.2727	0.0000
(>= 42)	100.00%	22.62%	33.67%	1.2923	0.0000
(>= 42.2)	100.00%	23.81%	34.69%	1.3125	0.0000
(>= 42.4)	100.00%	25.00%	35.71%	1.3333	0.0000
(>= 43)	100.00%	26.19%	36.73%	1.3548	0.0000
(>= 43.2)	100.00%	29.76%	39.80%	1.4237	0.0000
(>= 43.5)	100.00%	30.95%	40.82%	1.4483	0.0000
(>= 44)	100.00%	32.14%	41.84%	1.4737	0.0000
(>= 44.3)	100.00%	33.33%	42.86%	1.5000	0.0000
(>= 44.4)	100.00%	34.52%	43.88%	1.5273	0.0000
(>= 44.5)	100.00%	35.71%	44.90%	1.5556	0.0000
(>= 45)	100.00%	36.90%	45.92%	1.5849	0.0000
(>= 45.5)	100.00%	47.62%	55.10%	1.9091	0.0000
(>= 45.7)	100.00%	50.00%	57.14%	2.0000	0.0000
(>= 45.8)	100.00%	52.38%	59.18%	2.1000	0.0000
(>= 46)	100.00%	53.57%	60.20%	2.1538	0.0000
(>= 46.5)	100.00%	55.95%	62.24%	2.2703	0.0000
(>= 46.7)	100.00%	57.14%	63.27%	2.3333	0.0000
(>= 46.8)	100.00%	58.33%	64.29%	2.4000	0.0000
(>= 47)	100.00%	59.52%	65.31%	2.4706	0.0000
(>= 47.9)	100.00%	66.67%	71.43%	3.0000	0.0000
(>= 48)	100.00%	67.86%	72.45%	3.1111	0.0000
(>= 48.3)	100.00%	76.19%	79.59%	4.2000	0.0000
(>= 48.4)	100.00%	77.38%	80.61%	4.4211	0.0000
(>= 48.5)	100.00%	78.57%	81.63%	4.6667	0.0000
(>= 49)	100.00%	79.76%	82.65%	4.9412	0.0000
(>= 49.2)	100.00%	84.52%	86.73%	6.4615	0.0000
(>= 49.7)	100.00%	85.71%	87.76%	7.0000	0.0000
(>= 50)	100.00%	86.90%	88.78%	7.6364	0.0000
(>= 50.2)	100.00%	88.10%	89.80%	8.4000	0.0000
(>= 50.6)	100.00%	89.29%	90.82%	9.3333	0.0000
(>= 50.9)	100.00%	90.48%	91.84%	10.5000	0.0000
(>= 51)	100.00%	91.67%	92.86%	12.0000	0.0000

(>= 51.4)	100.00%	94.05%	94.90%	16.8000	0.0000
(>= 52)	92.86%	94.05%	93.88%	15.6000	0.0759
(>= 52.5)	78.57%	94.05%	91.84%	13.2000	0.2278
(>= 53)	64.29%	94.05%	89.80%	10.8000	0.3797
(>= 53.5)	64.29%	95.24%	90.82%	13.5000	0.3750
(>= 54)	64.29%	96.43%	91.84%	18.0000	0.3704
(>= 55)	57.14%	97.62%	91.84%	23.9999	0.4390
(>= 56.6)	50.00%	97.62%	90.82%	20.9999	0.5122
(>= 57)	42.86%	97.62%	89.80%	18.0000	0.5854
(>= 58)	21.43%	97.62%	86.73%	9.0000	0.8049
(>= 59)	7.14%	97.62%	84.69%	3.0000	0.9512
(>= 59.5)	7.14%	100.00%	86.73%		0.9286
(> 59.5)	0.00%	100.00%	85.71%		1.0000

Obs	ROC Area	Std. Err.	—Asymptotic Normal— [95% Conf.	Interval]
98	0.9647	0.0179	0.92969	0.99973

6 .
